# Parents’ perceived knowledge and beliefs on congenital malformations and their causes in the Amhara region, Ethiopia. A qualitative study

**DOI:** 10.1371/journal.pone.0257846

**Published:** 2021-11-02

**Authors:** Molla Taye

**Affiliations:** School of Medicine, College of Medicine and Health Sciences, University of Gondar, Central Gondar, Ethiopia; Shahjalal University of Science and Technology, BANGLADESH

## Abstract

**Introduction:**

Knowledge and beliefs of people on congenital malformations and their causes differ from society to society. As a result, there is a paucity of understanding community perceived knowledge and beliefs towards congenital malformations and their risk factors among children’s parents. Therefore, I sought to identify perceived knowledge and beliefs of parents on congenital malformations and their causes.

**Methods:**

An in-depth discussion and interview were carried out on purposively selected forty participants (women and men) in the Amhara region, Ethiopia. The data were collected from June to July, 2015. Semi-structured guiding topics/questions were used during the discussions and in-depth interviews. Note and audio records were taken while the participants discussed the topics. After the discussions and in-depth interviews were completed, the transcripts were read repeatedly to understand the participant’s words, phrases, ideas, and concepts. Then notes were taken to combine pieces of similar transcripts. I have employed thematic framework analysis. The relevant transcripts were scrutinized, labeled and coded manually based on their relevance to the objective of the study. Then the coded transcripts were determined and categorized according to the type of thematic variables.

**Results:**

The participants responded on three aspects of lived experience, perceived knowledge and beliefs on congenital malformations and their causes. Nearly half of the participants’ beliefs on the causes of congenital malformations were related to sin, contraceptive pills, un-prescribed drugs/medication use, and fertilizers (that is eating crops grown by using fertilizers). Almost all said that raising a child with a major congenital malformation was very difficult. About half of the participants’ belief on congenital malformations were traditional and resulted from poor awareness.

**Conclusion:**

The findings of the present study highlight the challenges and impacts of congenital malformations on parents who had children with and without congenital malformation. Lived experience, perceived knowledge and beliefs of children parents on congenital malformation and their causes can be helpful information for designing preventive actions. Hence, planning a preventive strategy and providing health education on congenital malformations and their causes for children parents are very necessary.

## Introduction

Knowledge and beliefs of people on congenital malformations (CMs) and their causes differ from society to society [1, 2]. CMs refers to any organ system malformation/anomaly that can be identified at prenatal period, during delivery or later in life. It could be a life-threatening condition, causing neonatal, infant, and child morbidity, mortality and long-term disability. Every year, about 3million children born with CMs [1]. In addition, more than 3million infant death occurs due to CMs globally each year [1]. CMs are also causes of embryonic and early fetal deaths [1, 2]. According to Bello et al., 2013 [2], around 95% of infant deaths occur due to CMs in low and middle income countries.

Many people believe that CMs occur to some families or people as an estrangement/punishment by God because of sin or sins, such as having poor faith and breaking societal norms, cultures, and the laws of God [1–3]. On the other hand, some societies also believed that CMs occur when a pregnant woman eats foods that are forbidden and has too many children [2]. Still other people believe that CM occurs when a pregnant woman is possessed by the devil. In addition, some people believe that CMs occur when a pregnant woman faces a dangerous event that threatens her fetus or has fears about something that threatens to bring bad results on her fetus [1, 4, 5].

Many societies have different views about CMs. For instance, in a study carried out on Kenyan women’s perceptions and attitudes about the causes of CMs, most of them said that when a woman conceived from a man other than her husband, used drugs, practiced family planning/birth control, contracted viral infections, had sexually transmitted diseases, failed to complete vaccines, and attending antenatal care, did heavy work, unfavorable position of fetus in the uterus, had hereditary problems, conceived from relatives, had extra marital sex, unfaithfulness, broke specific traditional taboos, broke taboos around sharing cooking places with grandmothers, had responsibilities for older people before they mature, cultivated someone else’s land, ate meat of pregnant cows, the way woman were created by God CMs resulted [1].

Similarly, as reported in a study conducted in Brazil on beliefs of mothers on risk factors associated with CMs, the respondent mothers said that when pregnant women were necklaces and bracelets, passed under wire fences or walked under ladders, spilled liquids on their belly or touched it with objects, carried key attached to their body, drank alcohol, smoked tobacco, used drugs, developed rubella infection, had consanguineous marriages, and were aged CMs developed [3].

According to some studies, many people have a wrong or poor level of knowledge and awareness about CMs [6–11]. However, any CMs can occur in any society, people, and race/ethnicity.

Parents who have knowledge and awareness about CMs can help/support their affected children and maintain family integration. Furthermore, children with CM may be confronted with discrimination and criticism. Teaching the society about CMs is a very important issue because it is a powerful tool to prevent societal discrimination and reviles. Understanding societal and parent’s knowledge, attitudes, perceptions/beliefs, and practices on CMs and their risk factors are important to uncover and address the problem to implement strategic plans for community teaching to eliminate or reduce the existing problems [4, 5, 12–15].

Understanding societal views, beliefs and perceptions towards CMs and their causes are also essential for public health actions. Again, it is also necessary to implement effective preventive strategies to reduce the occurrence and impacts of CMs. In Ethiopia, there seems to be limited knowledge and information on CMs. Thus, children with CM suffer from lack of care and medical treatment (for example surgical interventions). Similarly, the parents are mentally traumatized and stigmatized by their children’s problems. For many reasons, research on perceived knowledge, and beliefs and community practice relating to CMs and their causes have never been conducted in Ethiopia. Hence, the main objective of this study was to explore perceived knowledge, lived experience and beliefs of children parents on CMs and their risk factors. Therefore, this study can provide insights for policy makers. In addition, this study may be used as base line information for further studies on the problem.

## Materials and methods

### Study design

An in-depth discussion and interview were carried out on purposively selected participants. The study was conducted by using an exploratory phenomenological study design, which emphasis and focused on understanding and realizing the participant’s response word/s, phrases, ideas/concepts, beliefs and perceptions on CMs. A textual analysis method and interpretation were done after developing coded transcripts, themes and categories to explore the participant’s perceived knowledge and beliefs on CMs.

### Study sites

The study was conducted at Felege-Hiwot hospital, University of Gondar teaching hospital, Desse Hospital and Debark hospital. All of the hospitals are comprehensive specialized hospitals, except Debark hospital, which is a general hospital. All the hospitals provide a large number of various inpatient and outpatient services including delivery. The hospitals provide services by using its diagnosing devices, for example x-ray, CT scan, ultrasound and Magnetic Resonance Imaging. However, Debark hospital has ultrasound and x-ray devices only. Medical examination in the hospitals is carried out by radiologists, pediatricians, senior and other experienced medical doctors.

All of the hospitals are located in the Amhara regional state, Ethiopia. The region is subdivided into 12 administrative zones and three administrative cities. Besides, the region has 183 districts. In the region, there are 81 hospitals (5 comprehensive specialized, 3 general, 73 primary), 858 health centers and 3,560 health posts [16].

With an estimated total population of 20,399,004 (50.1% male, 49.9% female) [17], the Amhara National Regional State is the second largest region in Ethiopia. The capital city of the region is Bahir Dar, which is located in Northwest Ethiopia and 565 kilometers away from Addis Ababa, the capital city of Ethiopia. The Amhara region is the source of Lake Tana and Abay (Blue Nile) river. In the region, there are world heritage sites and tourist attraction places, such as Semien Mountains (including Ras Dejen Mountain), Lalibela and Fasil’s Palace (Fasil Ghebi). The majority of the people live in rural areas and the minority in large and small towns [17]. Most of the Amhara people are Christians with a good number of Muslims. Again, most of the rural inhabitants of the people are agriculturalist. Furthermore, nearly 61.8% of females and 76.8% of males were engaged in agricultural activities in 2016 [18]. The level of literacy rate for women and men were 44.9% and 65.7%, respectively [18]. Besides, 86.2% of females and 84.3% of males had no health insurance. As far as the wealth quintiles, 16.4%, 21.0%, 22.7%, 22.9%, and 17.0% of the people were lowest, second, middle, four and highest, respectively [18]. The total fertility rate was 3.7% in 2016 [18]. According to Central Statistics Agency of Ethiopia [18], 32.4% of mothers had no ANC follow up. However, about 67.1% of mothers received ANC from a skilled health care provider [18]. Astonishingly, home and health facility delivery rate were 71.1% and 27.1%, respectively [18]. Similarly, 4.4% of mothers weren’t assisted by any birth attendants during delivery, whereas 27.7% of mothers were assisted during delivery by skilled health care provider/birth attendant [18]. The total percentage of children whose births registered in 2016 by the health facilities were 1.3% [18].

### Study participants

The participants were parents/caretakers who were attending to their children under medical treatment and care at the study hospitals. Participants were purposively selected from four hospitals in the Amhara regional state, Ethiopia. Three-fourths of the participants were women aged 17–60 years, and one-fourth were adult men aged 24–65 years. In addition, three-fourths of the participants had children with CMs and one-fourth had no such children. The focus group discussion participants were government employees, private/self-employees, house wives, farmers, and un-employed people. The in-depth interviews also included two government employees, two house wives, two farmers, one self-employed, and one jobless participant.

### Selection of study hospitals

A total of four public hospitals were purposively selected and included in the study. The selected hospitals were Felege-Hiwot hospital, University of Gondar teaching hospital, Desse Hospital and Debark hospital. All the hospitals are tertiary leveled hospitals, except Debark hospital, which is a general hospital. The hospitals were selected on the basis of case load (CMs). All of the hospitals had various specialized units or departments, which provide medical services to adults, neonates and children. All the hospitals provide inpatient and outpatient services to patients (adult and children), pregnant mothers (antenatal care followers, including delivery care services) and family planning clients. In the study hospitals there are Obstetricians/Gynecologists, Pediatricians, Internists, Surgeons, Nurses, Midwives and other health professionals and para medical workers who are working permanently.

### Recruitment of participants

The study included forty participants who had children with and without CMs. A purposive sampling strategy was used to recruit participants from four public hospitals in the Amhara regional state, Ethiopia. The participants were approached in the children’s outpatient clinics, neonate units and pediatric wards. The purpose of the study was explained in detail to children’s parents and then invited to participate in the study. Then, volunteer participants were selected, and involved in the study. The selection of the participants was by considering the ability of participants to explain and provide substantive information or feelings about CMs. In addition, participants who agreed to participate and signed written informed consent were included in the study. All the participants were Amhara in ethnicity and fluently speak and listen Amharic language. In contrast, parents who weren’t voluntary and refused to sign written consent excluded in the study. Before the discussion began four focus groups and in-depth interviewees (8 individuals) were formed. Each focus group consisted of eight persons. The focus groups were formed based on the similarity of their background.

### Data collection method

The data collection procedure was in-depth semi-structured focus group discussions and interviews. The data was collected at public hospitals, while parents/caretakers were attending to their children’s medical care and treatment. Rooms were arranged at the hospitals by permission of hospital managers/directors to maintain privacy. Participants were identified and invited to participate in the study before FGDs and IDIs were carried out. The aim of the study was explained. After they agreed and gave written consents to participate, appointments were arranged. The FGDs and IDIs were conducted by using semi-structured topics (guiding) questions. The FGDs and IDIs began by asking participants some rapport creating questions. Once the study participants created a good rapport then the data collectors began to ask questions about the meaning or how they understood the expression CMs or birth defects, the causes, as well as perceived knowledge, lived experience and beliefs on the problem. Additional data were collected on the effects of substance abuse or behavioral factors, exposure to radiation, chemical substances, fertilizers, pesticides, heredity, folic acid, multivitamins, malnutrition/deficiency of micronutrients, and infections/diseases on embryos and fetuses. Data were also collected on sins/supernatural perceptions. The guiding topics prepared in English were translated to Amharic. The average time taken for IDIs were two hour and similarly the average time taken for FGDs were three and half hours. The FGDs were chaired by trained public health officers, assistant professors, midwives and nurses, and the face-to-face IDIs were conducted by the principal investigator. The FGDs and IDIs which were carried out until level of saturations were reached, which means no more ideas and concepts were added and taken in the form of written notes (by nurses and midwives) and audio recorded as participants talked about the points of discussion. The FGDs and IDIs were conducted from June-July, 2015.

To maintain the reliability and validity of the data collection, volunteer participants were selected by using a guideline, which help to decide the appropriate/suitable participants for this study. In addition, semi structured guiding questions were prepared. The topic of discussion was explained to participants to create a comfortable environment and enrich the data collection. The data were collected until it reaches saturation point. During the discussion the participants were treated with respect and assured by the investigator and facilitator of the discussions. Again, the participants were allowed and encouraged to explain their ideas, feelings/beliefs, perceptions and thoughts freely. Likewise, enough time was given for the interviews and focus group discussions. Besides, adequate sample size was determined for the interviews and focus group discussions. The texts were sorted, organized, coded and categorized to understand the information/meaning of the texts talked about or said by the participants. The texts were read more than two times to know the meaning/information of the texts. Then the principal investigator looked at the data again to understand what was said in the texts. Moreover, the transcripts were scrutinized to determine the refined themes and categories. An in-depth analysis and interpretation were carried out to describe the findings. Hence, the findings of this study are credible, conformable and can be generalized.

### Data management and analysis

The analysis was carried out after FGDs and IDIs were completed. The primary investigator developed a transcript based on the recorded audio and verbatim that captured all information obtained from participants. In the first place, the transcript was read repeatedly to understand the participant’s words, ideas, and concepts which were sorted and coded again according to the types of response. Deductive thematic framework analysis was used to analyze participant responses. Audio recorded information were transcribed verbatim. Then notes were prepared to combine pieces of similar words, ideas, and concepts together in order to make themes or categories. The themes that emerged from participants’ response on perceived knowledge, beliefs, lived experience and risk factors of CMs were recognized and organized into categories. The emergent themes and categories were identified by reading the transcripts many times and then coded. The coded transcripts were carefully scrutinized and refined at the time of analysis. The analysis was done by the primary investigator. Explanations and write ups were done on the refined transcripts.

### Ethical approval

Ethical clearance was obtained from the Institutional Review Board of Amhara regional Health Bureau Ethics Review Committee. Support letters were written to zonal health departments and study hospitals by health bureau. The purpose of the study was explained, and ethical clearance and support letters were shown to the participants. The study was carried out after permissions were obtained from hospital managers/medical directors, and written consents were obtained from the participants. Data collected from the participants were kept in safe and locked cabinets to maintain confidentiality.

## Results

Four FGDs (32 people) and eight (individuals) in-depth interviewees were included in the study. The participants responded on three aspects of lived experience, perceived knowledge and beliefs on congenital malformations and their causes. Twenty-eight of the participants were women aged 17–60 years and 12 were men with the age range of 24–65 years. The results of each theme are written as follows.

### Perceived knowledge on CMs

Both the FGDs and IDIs members had different understandings, ideas, and concepts on CMs and their causes. When the participants were asked about the expression “congenital malformations” or what they meant by them, the response (in FGDs) of a 46 years man (government employed), a 24 years man (Jobless), a 56 years man (farmer), a 40 years woman (self-employed) and in the IDIs a 33 years woman (house wife) were “A child born without a leg(s) or hand or arm”, whereas two men (44 and 51 years, both government employed) explained it as “Any visible abnormality on any body part/s, like spina bifida, cleft lip and palate, absence of phalanges or leg/arm”. On the contrary, 24 of the 40 participants (11 children’s parents had children with CMs and 6 children’s parents who had children without CMs) from each FGDs and 7 (5 women who had children with CMs and 2 men who had children without CMs) from the IDIs said, “I have no information on CMs…I don’t know the meaning of CMs”. However, eight participants who had children without CMs said, “To be honest, I have no idea…, but I guess it is a problem on body part/s….”

### Participants’ beliefs, lived experiences, and perceived knowledge on children with CM, which are likely to influence parents’ emotions at their child care duties are stated below:

A 38year old woman who had a child with spina bifida with hydrocephaly and bilateral clubfoot explained, “I was very frightened,…I was shocked, I thought I did something wrong, and my heart broke down when I saw my child…Then, I cried for several days…I was always confronting challenges in my life…and still I am in grief and thinking about the fate of my child”.

The response of another 33year old woman whose child had cleft lip and palate was, “I was sad and disappointed for many days…I didn’t know what to do…and I was hiding my child from people…but after I heard that the defect could be repaired by doctors, I hoped that my child could be treated and live as any normal child”.

A 40year old man who had a child with bladder exstrophy, cleft lip and palate said, “I was very sorry…stressful…I felt unfortunate, and hopeless…I kept thinking that my child couldn’t live, I did not expect that could happened to my child…” and he stopped talking. Again, another 35year old woman who delivered a child with frontal encephalocele at home, narrated, “I was worried…I was asking myself what it was and took my child to a nearby health center…Then the health professionals referred it to a nearby hospital, and they referred it to Zewditu Hospital, Addis Ababa. At that time, I felt that my child had no hope and couldn’t be helped”.

Many parents whose children had CM explained that the effects of their children’s problems put them in horrible and challenging condition in the following ways.

A 34year old woman whose child had gastroschisis said, “I was always thinking that my child was going to die…my thoughts were about my child dying…and I was desperate…I was in shock…I have had no hope since then…”. Similarly, another 55 year old man whose child had cleft lip and palate went on: “I was afraid…I didn’t think my child was going to live, I was traumatized, but now I hope my child’s problem can be repaired by health care providers; hence, my child will survive and grow, because I was considering that the child would die due to feeding problems… and that it would be isolated when it grows up due to discrimination”.

A 27year old woman who had a child with open spinal bifida stated, “I felt anxiety, panic, and cried…I was always sad and felt that my child had no hope and would die”, and asked, “Why doesn’t the government find a solution for such kinds of congenital malformations?”.

Most parents (mothers and fathers) of children with CMs said, “The problem is very distressing, causing grief, shame…We felt as if we were cursed and apathetic”. In addition, parents stated: “We not only suffer from psychological conditions but also face economic crises…We spent a lot on care and treatment of the child…We leave home and worry about the other children we leave at home”.

### Beliefs on causes of congenital anomalies

With regard to the causes of CMs, participants’ general beliefs were varied. When all of the participants were asked what the causes of CMs were, eighteen (16 women who had children with CMs and 2 men who had children with CM) of the participants said, “I have no knowledge of the causes of CMs …, I don’t know exactly the causes of CMs”, while twenty (17 women who had children with CMs and 3 men (two men had children with CMs and one man who had a child without CM)) out of all FGDs and IDIs believed that sins/spiritual consequences, use of contraceptive pills and/or un-prescribed drugs, and eating fertilized crops caused CMs. Only two individuals (a 33years old who had child with CM and 45year old woman who had child with CM believed and related the causes of CMs to heredity and behavioral factors, such as alcohol consumption and smoking cigarettes.

Further discussion points/questions were raised to assess participants’ beliefs and knowledge on the causes of CMs. The majority (34/40) of the participants (twenty-two women who had children with CMs in the FGDs and IDIs and all men who had children with and without CMs in the FGDs and IDIs) explained, “Alcohol drinking during the first three months of pregnancy would not have any effect on the embryo/fetus”, while two women (a house wife and a farmer) who had children with CMs said, “I don’t know the consequences of alcohol”, and six participants (three government employees men and two self-employees men, and one 32 year old government woman) who had children with and without CMs stated, “Alcohol consumption would cause addiction, liver disease, and mental retardation”. In addition, twelve women (11 had children with CMs and one child without CM) and eight men (6 had children with CM and 2 children without CM) said, “I knew that abusing alcohol by women and men led to addiction and liver disease but I didn’t hear from health professionals and the government about the effects of alcohol on unborn babies, as a result I did not consider alcohol drinking during pregnancy had consequences on embryos and fetuses”. On the other hand, a 55year old man who had a child without CM in the IDIs said, “CM could occur when a drunken and intoxicated husband shouted at his pregnant wife making her extremely worried”. This participant also believed and said, “Sunlight deficiency could cause congenital malformation”. Another 35 years old man who had a child with CM in the FGDs explained, “If a pregnant woman doesn’t make antenatal care visits, is not vaccinated for poliomyelitis, tetanus, and measles her baby would be born with CM”. Very few (a 34 year old man who had child without CM and a 31 year old woman who had child with CM) participants said, “If a pregnant woman desired to eat or drink something particular but failed to get what she wanted, the baby would be born with a CM”.

More than half (29 of the 40 women and men, who had children with and without CMs) of the participants pointed out, “Evil spirits or ghosts or fear that anguish a pregnant woman would lead her to having a baby with a CM”. Moreover, few (4 women who had children with CMs and one man aged 60 years who had no child with CM and another aged 30 years who had a child with CM) participants said, “Babies with CMs might be born to persons who committed crimes, and did not respect religious codes”.

A 40 year old man who had child with CM thought, “Use of un-prescribed drugs could result in deafness, blindness, and inactiveness”, whereas seven men (4 had children with CMs and 3 had children without CMs) and six women who had children with CMs stated, “Un-prescribed drug use by pregnant women could cause miscarriage as well as mental retardation and disorder/CM in babies”. In contrast, some participants (farmers, house wives, jobless and self-employee) who had children with CMs said, “I didn’t know …., didn’t hear about specific effects of drug use during pregnancy on embryos/unborn babies”. On the other hand, twenty-four participants (sixteen women and eight men who had children with and without CMs) among all FGDs and IDIs said, “Drugs poorly handled were being sold without prescriptions by every drug vender/store;…this, people were using drugs unnecessarily, and that could affect the health of people, especially pregnant women and their unborn babies. Therefore, concerned principal authorities must control drug venders and stores which sell drugs without prescription/s”.

Seventeen participants (3 government employees, 2 self-employees, 4 house wives, 5 farmers, and 3 jobless) who had children with CMs explained, “Cigarette smoking would lead to lung disease in babies after birth”. However, more than half (21 women who had children with CMs and 2 men (one had child with CM and the other had child without CM)) of the participants said, “I had no information…I didn’t know the effects of cigarette smoking on the embryo/fetus”. In addition, this participants by occupation were 7 farmers, 6 house wives, 5 jobless, 3 self-employees, 2 government employees.

Fourteen participants (ten women who had children with CMs and four men (3 had children with CMs and one had child without CM) in each occupation groups believed and said, “Radiation, chemicals, and pesticide exposures would harm the fetus, resulting in unhealthy babies”, whereas six participants who had children with CMs stated, “I didn’t know their effects on embryos/unborn babies”. In contrast, half (20/40) of the participants with and without CMs (both women and men) in all FGDs and two (one woman aged 60 years who had no child with CM and one man aged 65 years who had child with CM) in IDIs explained, “Radiation is good during pregnancy and has no effect on the embryo and fetus, rather it helps to identify the disease of the mother and the situation of the fetus in the womb; it won’t affect the fetus”.

Each of a few (4/40) participants who had children with CMs (27 years (female), 34 years (male), and 45 years old (female) in FGDs and one woman (46 years) who had child with CM in the IDIs) said, “I have no information/knowledge…so I don’t know the specific effects of nutrition/micronutrient deficiency/folic acid/iron folate deficiency on the embryo, whereas the majority (30/40) of the participants who had children with CMs reported, “It would have a direct effect on the health of the baby and the mother, except that the baby would be thin and underweight”, while few (two women participants, that is one house wife and one farmer who had children with CMs) stated, “Mothers would be thin and die due to starvation”. However, two women (government employees and who had no children with CMs) participants pointed out, “If a pregnant woman doesn’t take folic acid/iron folate CM might occur to her unborn baby”. On the contrary, the majority (in each occupation) of the participants didn’t consider folic acid/micronutrient deficiency or malnutrition as causes of CMs.

Diseases/infections, especially sexually transmitted diseases, like syphilis, and vaccine preventable diseases, such as tetanus, and measles were cited as the causes of CMs by two participants (two women government workers, whose age were 27 and 45 years). However, three (a house wife and two farmer who had children with and without CMs) participants (one woman aged 28 years and one man aged 42 and a 60 years male (who had no children with CMs)) said, “Diseases/infections would not cause CMs”.

## Discussion

Perceived knowledge and beliefs on CMs and their causes were varied among the participants of this study. Some of the participants poorly understood CMs and their causes, while a few participants had little knowledge on CMs and their causes. On the contrary, the majority of the participants had no knowledge on CMs and their causes. This finding was closer to the findings reported in Ghana [2], Kenya [1], and Iran [6]. This indicates that societies shared beliefs, and perceived knowledge on CMs as mysterious circumstances. This may be due to the influences of religions, cultures, and societal norms as well as educational backgrounds and lack of information about CMs. However, the findings of the present study were slightly higher than to those studies conducted in Brazil [3] and Egypt and Saudi Arabia [4]. In addition, according to studies, perceived views of different societies on the causes of CMs were widely varied among countries, though, sometimes there might be overlapping opinions and views across societies [9, 13–15, 19–27].

As far as literature is considered, perceived knowledge and beliefs of different societies on CMs and their causes might overlap or differ due to educational status and life-experiences [1–4, 6, 7]. For example, a study carried out in Kenya suggested that cultural beliefs on the etiology of CMs and adverse pregnancy outcomes were associated with particular supernatural impressions or perceptions deep rooted in the community [1]. Likewise, a study conducted in Brazil on the causes of CMs indicated that 1,191 of the responses to 3,219 interviews were related to beliefs, myths, or superstitions [3]. Another study carried out in Ghana on Ghanaian pregnant mothers’ knowledge about CMs showed that about 48.1% of participants held that the causes of CMs could be supernatural factors [2].

In the current study, some of the participants believed that contraceptive/s could cause CMs. This finding was closer to that of a study conducted in Kenya [1]. Furthermore, some of the participants of this study explained that folic acid/iron folate deficiency could lead to CMs. This finding was similar to the findings of other studies [4, 7]. Moreover, many studies suggested that iron folate prevents/reduces the occurrence of certain types of CMs, for example, neural tube defects [5, 12, 28–33]. This indicates that few participants were aware that folic acid/iron folate protects from some CMs.

In this study, the majority of the participants agreed that alcohol had no effect on unborn babies except causing addiction, mental problems, and diseases, such as liver disease on the mother, while some participants said that they did not know the effects of alcohol on the embryo/fetus. This finding was different from those of other studies. This could be due to lifestyle experiences or differences in educational backgrounds of the study participants or may also be due to perception and cultural differences.

According to the beliefs and views of the participants, the consequences of un-prescribed drug use by pregnant women were miscarriage, delays in mental developmental, and CM. This finding was in line with that of other similar studies [1–3]. In addition, one participant of the present study said that in active babies, deafness, and blindness are consequences of un-prescribed drug use by mothers during pregnancy.

The participants suggested that cigarette smoking mainly caused lung disease in the mothers and babies after birth rather than CMs. On the other hand, some of the participants had no knowledge on the effects of cigarette smoking as a cause of CM on the embryo. This finding was disagreed to other similar study [2]. This could be due to low level of knowledge about the effects of cigarette as a cause of CM among our participants. Actually, in Ethiopia, most women do not smoke cigarettes, especially rural women, due to religion, cultural, and social norms.

In the present study, just a few participants believed that diseases/infections, including sexually transmitted once, like syphilis and others, such as measles, contracted by the mother during pregnancy, particularly early pregnancy, could lead to CMs. This finding is similar to those of other studies [1, 4].

The participants of this study suggested that radiation and chemicals could harm both the mother and the fetus; the unborn baby would have a higher health risk after birth. In addition, the majority of the participants of this study said that radiation had advantages for identifying diseases of mothers and to recognize the position of the fetus in the womb. On the contrary, few participants lacked knowledge about the effects of radiation and exposure to certain chemical agents and pesticides. However, other studies identified radiation as a cause of CMs [4, 6]. Fertilizers (i.e. eating crops grown by using fertilizers) were explained as causes of CMs by the participants, but this was not discussed in other similar studies. This could be due to differences in personal views (opinions) and educational backgrounds.

A few participants of this study argued that heredity had a role in the causation of CMs. Nevertheless, the majority of the participants believed that heredity had no effect on CMs. In contrast, some of the participants did not know the effects/impacts of heredity on embryos/fetuses. In other similar studies respondents mentioned heredity as a cause of CMs [1, 4, 7].

In the present study, some of the participants’ responses on nutritional deficiency/malnutrition were contradictory to each other. The majority stated that it has direct effects on the fetus, causing it to become thin and underweight. In addition, they said the mother would be thinner than usual and probably die due to starvation/hunger. On the contrary, two participants of this study believed that CM could occur to the baby when a pregnant woman wants some food item/s or drinks (i.e. to eat or drink) and fails to get them. On the other hand, a study conducted in Ghana reported that about 23.7% of the respondents believed that CMs could occur because of eating some forbidden foods during pregnancy [2].

In this study, a few participants believed that not attending antenatal care and vaccinating for preventable illnesses, such as tetanus, measles, poliomyelitis, and others during and before pregnancy were forwarded as possible causes of CMs. Such opinions were also reflected in other similar studies [1].

In the present study, the majority of the respondents stated that most of the society believed that the causes of CMs were punishment by God due to estrangement from God or sins committed by affected children’s parents/families considered as cursed and evildoers by the community. However, some participants opposed these opinions. This indicates that social impressions and beliefs on CMs were contradictory to each other or mixed up. In addition, this shows that the community had no adequate information on CMs and their causes; as a result, there were different views which needed health education or genetic services. However, there has been no community genetic service/s in Ethiopia. This finding is in line with those of other similar studies [1, 4].

All of the participants of this study considered CMs as extremely challenging and terrible conditions. Therefore, I believe that the present study can provide valuable information about community beliefs and perceived knowledge on CMs and their causes is essential for public health actions.

The findings of this study could be interpreted with the following limitations. The present study participants were government, non-government, self-employees, farmers, house wives and jobless, as a result, this might introduce some bias. The reason for not including religious leaders, traditional birth attendants, health extension workers, and expert health professionals on CMs and their risk factors were due to that the study was conducted in the health facilities.

In conclusion, this study highlights the stressful challenges and impacts of CMs on parents who had children with and without CM. Therefore, understanding community perceived knowledge and beliefs towards CMs and their etiologic factors are very necessary to implement effective preventive strategic plans, to educate the people, and to create awareness that could help to reduce the occurrence and impacts of the problem. Hence, the present study can give insight to health care providers, policy makers, and health service managers so that they could play their roles by fighting against CMs.

## Abbreviations

CM: congenital malformation
CMs: congenital malformations
FGDs: focus group discussions
IDIs: in-depth interviews
